# Unveiling the gut-eye axis: how microbial metabolites influence ocular health and disease

**DOI:** 10.3389/fmed.2024.1377186

**Published:** 2024-05-10

**Authors:** Yvonne Nguyen, Josephine Rudd Zhong Manis, Nicole Marie Ronczkowski, Tommy Bui, Allston Oxenrider, Ravirajsinh N. Jadeja, Menaka C. Thounaojam

**Affiliations:** ^1^Mercer University School of Medicine, Macon, GA, United States; ^2^Departments of Cellular Biology and Anatomy, Augusta University, Augusta, GA, United States; ^3^Biochemistry and Molecular Biology, Medical College of Georgia at Augusta University, Augusta, GA, United States

**Keywords:** microbiome, bile acids, butyrate, ocular diseases, short chain fatty acids

## Abstract

The intricate interplay between the gut microbiota and ocular health has surpassed conventional medical beliefs, fundamentally reshaping our understanding of organ interconnectivity. This review investigates into the intricate relationship between gut microbiota-derived metabolites and their consequential impact on ocular health and disease pathogenesis. By examining the role of specific metabolites, such as short-chain fatty acids (SCFAs) like butyrate and bile acids (BAs), herein we elucidate their significant contributions to ocular pathologies, thought-provoking the traditional belief of organ sterility, particularly in the field of ophthalmology. Highlighting the dynamic nature of the gut microbiota and its profound influence on ocular health, this review underlines the necessity of comprehending the complex workings of the gut-eye axis, an emerging field of science ready for further exploration and scrutiny. While acknowledging the therapeutic promise in manipulating the gut microbiome and its metabolites, the available literature advocates for a targeted, precise approach. Instead of broad interventions, it emphasizes the potential of exploiting specific microbiome-related metabolites as a focused strategy. This targeted approach compared to a precision tool rather than a broad-spectrum solution, aims to explore the therapeutic applications of microbiome-related metabolites in the context of various retinal diseases. By proposing a nuanced strategy targeted at specific microbial metabolites, this review suggests that addressing specific deficiencies or imbalances through microbiome-related metabolites might yield expedited and pronounced outcomes in systemic health, extending to the eye. This focused strategy holds the potential in bypassing the irregularity associated with manipulating microbes themselves, paving a more efficient pathway toward desired outcomes in optimizing gut health and its implications for retinal diseases.

## 1 Introduction

The multifaceted roles of gut microbiota (GM) extend beyond conventional understanding, encompassing a spectrum of physiological and pathological functions (1–4). Beyond their role in digestion, these microorganisms communicate with various organ systems, prompting a paradigm shift in comprehending the interconnectedness of bodily functions (1–4). This review investigates into the evolving role of GM’s influence on ocular health, challenging historical presumptions of organ isolation and unveiling a profound interplay between microbiota-derived compounds and ocular pathogenesis. Recent scientific advancements have reshaped our perception of organ functionality, emphasizing the substantial impact of GM and their metabolites, notably short-chain fatty acids (SCFAs) like butyrate (5–7) and bile acids (BAs) (8–10), on various diseases including ocular pathologies. These observations contradict traditional beliefs of organ sterility, marking the GM as a key influencer in ocular pathology. This paradigm shift necessitates reevaluating therapeutic approaches within ophthalmology, highlighting the pivotal role of the GM in ocular diseases. The dynamic nature of the GM, thriving with diverse microbial communities and their metabolites, coordinates a complex interplay within the gut and extends its influence to ocular well-being (11). Understanding this bidirectional communication between the GM and ocular health propels exploration into potential therapeutic avenues embedded within this nexus. Acknowledging the promising therapeutic potential in manipulating the GM and its metabolites, a transformative shift advocates for a precise, tailored approach over broad interventions. This exemplary change comprises the utilization of specific microbiome-related metabolites as targeted therapeutic strategies, diverging from the historical blanket solutions (12–14). This nuanced precision aims to harness the potential of microbiome-related compounds, particularly within the context of retinal diseases, catering to individual microbiome intricacies. By advocating for a focused strategy tailored to the intricacies of individual microbiomes, this review aims to expedite and magnify outcomes in optimizing gut health and its implications for ocular pathologies. This precision-driven approach offers a more efficient pathway toward desired therapeutic outcomes, steering away from the uncertainties associated with altering the entire microbiome landscape.

## 2 The gut microbiota

The intestinal tract is inhabited by a diverse array of microorganisms, commonly referred to as the GM. The GM has recently come to light as a major topic of investigation in a multitude of research fields. Though it is debated when gut microbial colonization occurs (15, 16), it is widely accepted that there is a vast variety of microbial species within and on the surface of the body. These microorganisms include viruses and bacteria, among other microbes, which have physiologic and pathologic functions that affect many organ systems. Once present in the gut, these bacteria communicate with the rest of the body via various mechanisms, and their symbiosis and possible dysbiosis have been correlated with health and disease states in many different organ systems, ranging from the skin to the liver to the eye (17–19). This dynamic community has a wide range of impacts on our lives and is a major contender in altering health outcomes. The role of the GM should be considered in all fields of study, even in Ophthalmology, where the paradigm of organ sterility is constantly challenged (20).

### 2.1 Development of GM during fetus and postnatal

Shortly after delivery, the gastrointestinal tract of a newborn is colonized by bacteria (21). Throughout this process, there are many factors that can alter the neonatal microbial community, including birth mode, environment, timing, diet, and gestational complications. In the neonatal gut, bacteria are less abundant but more diverse than those within the mature gut, most likely due to lower biomass and decreased species competition. The lower biomass complicates sampling of the neonatal gut, and there is a lack of consensus on the species present. However, *Proteobacteria* and *Actinobacteria* seem to dominate (22, 23), though *Lactobacillus species* (phylum *Firmicutes*) are commonly present as well (24). By the age of three, the gut bacterial population stabilizes and resembles that of adulthood (25). The adult gut consists of a highly individualized community that is impacted by extrinsic and intrinsic factors, including diet, environment, hygiene, host genetics and epigenetics, immune changes, metabolic status, and medications. In adulthood, the major phyla that colonize the gut are *Bacteroides* and *Firmicutes,* with anaerobes being present in the greatest quantities due to the gut’s limited oxygen availability (26, 27).

**Figure 1:**
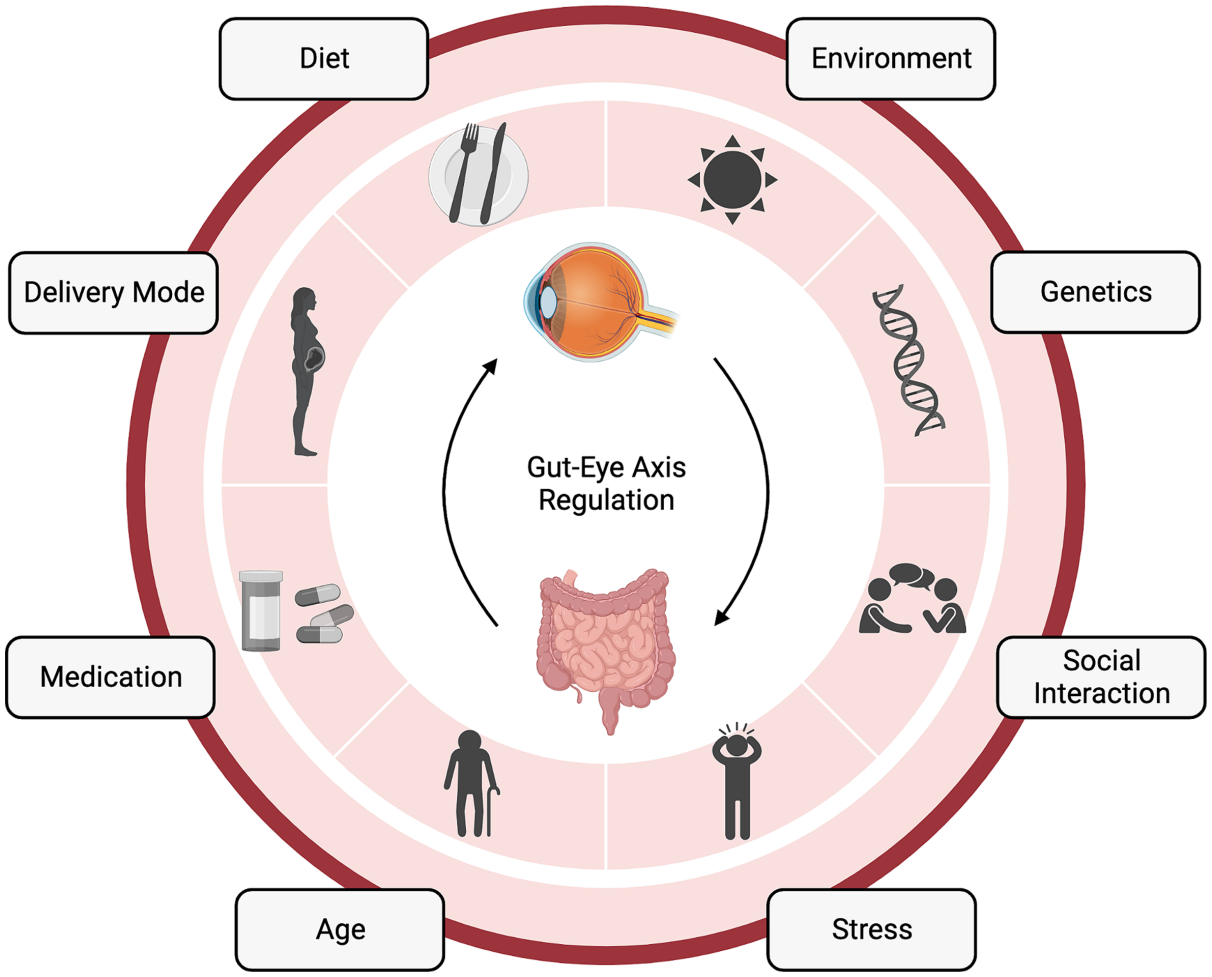
The gut-eye axis in retinal health and disease. The complex interplay between the gut microbiome (GM) and retinal health. This diagram illustrates the numerous factors influencing the gut-eye axis and its potential role in various retinal diseases. “Created with BioRender.com”.

The prenatal period and the baby’s delivery mode, i.e., cesarean or vaginal, shape the neonatal microbiota as it is the baby’s first exposure to the world full of microbes (Figure 1). During the prenatal period, the baby is susceptible to microbiota changes, specifically due to pregnancy complications (28). It has been shown that the neonate takes on more of the maternal skin microbiome (e.g., *Staphylococcus*, *Corynebacterium*, and *Propionibacterium*) in cesarean delivery, and vaginally delivered babies take on the maternal vaginal flora (e.g., *Lactobacillus*, *Prevotella*, and *Sneathia*) (29). Other groups have found different changes in the microbiota based on birth mode (30, 31), but it is important to note the potential for microbiota alteration through factors outside of the delivery mode itself, such as antibiotics delivered to moms undergoing C-section, vaginal swab from mom given to C-section baby, or birth environment. For example, Selma-Royo in 2020 found that place of birth (i.e., home vs. hospital) was the most significant driver of the neonatal microbiota when compared with other birth alterations (32). Lastly, younger gestational age at the time of birth has been correlated with an immature GM and can even alter the composition of bacterial metabolites, such as SCFA present (25).

From the immediate neonatal period and throughout infancy, the diet of both mom and baby can impact the bacterial community. Neonatal diet, i.e., breastfeeding or formula feeding, and feeding frequency and duration, can alter the availability of breast milk components to the baby (Figure 1). Breast milk contains not only nutrients for the baby but also antimicrobial peptides, human milk oligosaccharides, and potentially bacteria (33). Importantly, maternal fat and fiber consumption alter the breast milk and, therefore, the baby’s microbiota (25). Breastfeeding also provides an opportunity for the neonate to interact with the maternal skin microbiome and ingest these bacteria. Upon feeding, the baby is inoculated with factors that shape their intestinal microbial community and the metabolites it is able to produce (34). The baby’s transition to solid food also induces a microbiota shift those further shapes the microbiota and prepares the baby for adult immune insults (35) (Figure 1).

### 2.2 Ocular surface microbiome

The ocular surface, comprising the conjunctiva, cornea, and eyelids, hosts a diverse array of microorganisms. Recent investigations have revealed the intricate microbial communities present in this environment, shaped by constant exposure to the external world. Among the prominent bacterial genera are *Staphylococcus*, *Corynebacterium*, *Streptococcus*, *Propionibacterium*, and *Micrococcus* (36). These species have evolved mechanisms to evade tear film defenses and interact with the local immune system. Dysbiosis of the ocular microbiota can occur due to factors like antibiotic use, disease states, and gut microbiome alterations. Notably, these microbiotas play a role in immunity, inflammation, and various eye conditions, including conjunctivitis, blepharitis, keratitis, and dry eye syndrome (37). Understanding their impact is crucial for maintaining ocular health and guiding therapeutic strategies.

### 2.3 Factors affecting microbiome

Factors both intrinsic and extrinsic to the host shape the GM throughout our lifetimes (Figure 1) (38). Certain gene loci have been attributed to microbiota heritability (39), and the microbiota can even alter host epigenetics via metabolites (40), suggesting a bidirectional host-symbiont genetic relationship. Another bidirectional relationship that is intrinsic to the host is immunity, which is both shaped by gut bacteria and can shape the bacteria’s survivability in the gut (41). Additionally, the metabolic status of the host can be shaped by bacteria (42), which, can also be considered a symbiotic relationship between the host and gut community. One extrinsic factor that can shape the GM is antibiotics. In terms of the GM, antibiotics, by nature, kill beneficial microbes. However, there are still studies claiming their utility, specifically in terms of the gut-eye axis. Oral antibiotics have been shown to reduce the severity of experimental autoimmune uveitis in mice by altering the composition of the GM, raising the frequency of regulatory T cells (Treg) in the intestine, and decreasing inflammatory cytokines (43).

In contrast to antibiotics, probiotics or fecal transplants represent methods of introducing microbiota to a host GI tract that have been the subject of great investigation. These methods function by directly inoculating the GI tract with exogenous microbes. Probiotics, by definition, are a strain of bacteria that have been shown to have at least one clinical benefit in a peer-reviewed study (44). However, their lack of regulation in production and sale led to mixed reviews. On one hand, many studies claim their benefit in many dysbiotic states or for general preventative medicine (45, 46), however, without proper education and regulation, their benefits are quite limited in practice. For example, there are no guarantees the bacteria strain in the probiotic is alive at the time of reaching the consumer, much less reaching the GI tract where it should be exerting its benefit [reviewed by Ayichew et al. (47)]. Fecal transplant, however, is a regulated process that has shown very promising results, for example, in the treatment of *C. difficile* infection (48). Though due to its risk for infection or contamination, this is a very costly and scarce procedure (49).

Another extrinsic factor of great interest for microbiota is the diet. Generally, the regional basis of diet (i.e., Mediterranean, Western, etc.) and these tendencies can greatly influence the overall profile of bacteria in the gut via their macronutrient composition. Carbohydrates, for example, can alter the SCFA production of the bacteria, while fats and proteins can foster the growth of specific bacterial species. Dietary fiber, which is non-absorbable to the host, can feed the gut bacteria and push contents through the GI tract, facilitating movement and a dynamic gut community (50). Outside of nutrition, factors such as host hygiene and social isolation can alter the GM (51). Even urbanization and changing host environments, such as living in a rural or urban area, can alter microbe exposure and determine which species are present in the host diet and community (52). Several studies have found reduced gut microbial diversity and altered microbiota composition in adults with obesity compared to normal-weight adults (53). In one study, weight loss in 33 adults with obesity was associated with an increase in gut microbial diversity and in butyrate producing GM (54). Similarly, levels of *Bacteroides fragilis* and *Lactobacillus* were higher in adolescents who lost more weight while on the same calorie-restricted diet than those who lost less weight (54). In addition to the overall dietary profile, adding so-called prebiotics (i.e., those fermented foods that might feed microbes) has been shown to be associated with improvements in metabolic outcomes and gut barrier against pathogens (46). Overall, diet plays a major role in shaping gut outcomes and could very well play a role in systemic and eye pathologies through the axes described in this review. Lastly, medications, in addition to the diseases they are being used to treat, are an important mix of iatrogenic and intrinsic sources of GM variability (55).

## 3 The gut-eye axis

Although the precise mechanisms of communication between the GM and the eye are still being studied, it is becoming increasingly evident that GM and its products can impact ocular health, showed by its numerous systemic functions (Figure 1). In this review, we aim to uncover potential ways the GM may be in communication with the eye and specifically highlight how this might be altered in dysbiosis in various retinal ailments. As discussed above, the GM communicates with the rest of the body in a multitude of ways. Additionally, it is apparent there are opportunities for the so-called gut-eye axis to come about, whether that be through immune modulation, metabolite communication, or direct connections, as happens in the gut-brain axis. Furthermore, the GM may modulate immune responses that play a role in age-related macular degeneration (AMD) pathogenesis, including inflammation and oxidative stress. Taken together, this highlights the importance of GM in bidirectional immune modulation and the potential for its role in ocular disease. Emerging research suggests that GM and its metabolites may have implications for ocular health, and we will highlight this intricate relationship below.

Systemically, inflammation can result from alterations in the GM and its metabolites. While eye diseases are usually associated with an infectious component, eye inflammation due to GM dysbiosis is another way to develop eye disease (56). This process can be transient until the leaky gut tight junctions are healed, or more permanent, as seen in IBD, which presents with extraintestinal manifestations including ocular may be due to gut bacteria triggering a systemic, adaptive immune response (57). Thus, leaky gut, caused by diseases that breach the intestinal barrier, such as Crohn’s, as well as lifestyle risk factors inducing inflammation, such as smoking or alcohol consumption, plays a key role in establishing the gut-eye axis through systemic spread of bacteria and digestive products (58). Other methods of communication have been shown to play an important role in the gut-eye axis and ocular disease development, including metabolites and immune factors. Inflammation caused by activation of T-cells and breach of the blood-retina barrier is seen in autoimmune uveitis (AU), while immune activation due to gut bacterial irregularities due to high-fat diet is also seen in AMD in mice (59). The blood-ocular barrier consists of both the blood-aqueous barrier and the blood-retinal barrier (BRB) (60). The BRB is tight and restrictive and has inner and outer components. The outer component consists of tight junctions between retinal pigment epithelial cells, and the inner component consists of tight junctions between retinal capillary endothelial cells (61, 62). The BRB acts as a physiologic barrier from the systemic circulation that regulates ion, protein, and water flux into and out of the retina and is vital to maintaining the eye as a privileged site within the body (60). The microenvironment of the retina must be tightly regulated because the retina is susceptible to oxidative stress, which would cause damage to the central vision. The tight junctions of the blood-aqueous barrier are leaky, whereas the tight junctions of the BRB are nonleaky (63). The BRB has properties similar to the BBB, but small protein tracers like microperoxidase (19 kDa) are capable of entering the BBB but not the retina (63). This indicates the intercellular junctions of the retinal endothelium are sealed more tightly, most likely due to a large amount of zonulae occludent that forms the bulk of the BRB (63). Along with the physical barrier that the BRB provides, it also is an environment with active mechanisms of immunoregulation and immunosuppression (60). Neuropeptides in the aqueous humor assist in suppressing induction of delayed-type hypersensitivity and induce regulatory immunity. Collectively, the neuropeptides suppress the activation of Th1 cells while promoting the induction of CD25+ and CD4+ regulatory T cells (64). Inflammation can lead to breakdown of the BRB, leading to autoimmunity and inflammation and would also allow systemic drugs to penetrate into the eye (60).

Aging is another key factor in creating the gut-eye axis by weakening physical barriers and immune regulatory signals and increasing permeability, both in the retina through weakening of the aforementioned three tissue layers that give the eye immune privilege and in the intestines (56). Other factors that can play a role in gut-eye axis development include epigenetics, dietary influences, and GM metabolites (65). Dietary influences on the gut-eye axis have been subject to much investigation. Intermittent fasting has been shown to both restructure the GM as in the gut-brain axis and reduce risk factors for certain ocular diseases like blood pressure and heart rate (66, 67).

Bacteria themselves also influence susceptibility to ocular disease, as occurs in bacterial keratitis, where altered species in the GM increase corneal inflammation (68). Recently, new evidence through genome-wide association studies has emerged that metabolites of the GM, such as SCFAs, can further modify the epigenome and trigger intraocular inflammation. Kim et al. investigated the modulating effects of IRT-5, a cocktail of five probiotic strains, and discovered the treatment prevented the development of experimental autoimmune uveitis (EAU) and attenuated clinical manifestations of autoimmune dry eye models (69). In glaucoma, an imbalanced gut marked by high *Firmicutes/Bacteroidetes* ratio and pro-inflammatory bacteria like *Prevotella* worsens neurodegeneration, as evidenced by studies in germ-free mice (70). Higher levels of SCFAs, such as propionate, acetate, and butyrate, were further associated with glaucoma patients with a GM high in Dysgonamonadaceae species (71). AMD presents no single microbial signature, but specific shifts like increased *Prevotella* and *Holdemanella* point toward potential risk factors (72). High-fat and high-glycemic diets exacerbate AMD by altering the microbiome and promoting inflammatory markers (72). Uveitis patients exhibit a gut lacking diversity and are enriched in pro-inflammatory bacteria, with depleted butyrate-producing bacteria crucial for gut barrier function and anti-inflammatory response (73). Studies employing antibiotics and experimental models suggest the GM directly influences the severity of uveitis (73). Interestingly, mouse models of inherited retinal degenerations like retinitis pigmentosa (RP) and Batten disease exhibit altered gut bacterial profiles compared to healthy controls. In these models high-fat diets worsen retinal degeneration by further altering microbial diversity and inflammatory processes (74), suggesting potential avenues for managing these devastating conditions through microbiome modulation. Retinal vascular diseases like diabetic retinopathy (DR) and retinal artery occlusion show lower bacterial diversity and specific microbial shifts linked to inflammation and cholesterol metabolism. Microbial metabolites like lipopolysaccharides and trimethylamine N-oxide may play a role in disease development through vascular risk factors (75). Preterm infants with retinopathy of prematurity (ROP) often exhibit decreased *Firmicutes* and *Lactobacillus* with increased *Enterobacteriaceae*, potentially impacting vascularization and contributing to oxidative stress (76). An analysis of GM in premature infants by Skondra et al. in 2020 showed changes in early GM composition associated with severe ROP and proposed metabolic pathways as potential therapeutic targets (77). Understanding the microbiome-ROP relationship could advance screening and intervention strategies. Similar to the gut, a diverse and balanced ocular surface microbiome is key to a healthy eye. Various eye diseases, from dry eye to Sjögren’s syndrome, are characterized by a depletion of beneficial bacteria and an overgrowth of harmful ones, disrupting the delicate balance and contributing to disease development (78) (Table 1).

**Table 1:** Specific microbiota changes and ocular pathologies.

Organism(s)	Ocular disease	Findings	References
Veillonellaceae, Ruminococcaceae, Lachnospiraceae, Clostridiaceae, Lactobacillaceae, Turicibacteraceae, Peptococcaceae, and Gemellaceae	Bacterial keratitis	Eight families from the phylum *Firmicutes* were higher in abundance in humans with bacterial keratitis compared to controls	(68)
Firmicutes, Bacteroidetes, and *Prevotella*	Open-angle glaucoma	High *Firmicutes/Bacteroidetes* ratio and *Prevotella* worsen neurodegeneration in studies with germ-free mice	(70)
*Dysgonamonadaceae* species	Open-angle glaucoma	Higher levels of short-chain fatty acids were also associated with gut microbiota high in *Dysgonamonadaceae* species	(71)
*Prevotella* and *Holdemanella*	Age-related macular degeneration	Increased *Prevotella* and *Holdemanella* are potential risk factors for Age-Related Macular Degeneration	(72)
Clostridiales order and Bacteroidales order	Age-related macular degeneration	Increased *Clostridiales* in mice with features of age-related macular degeneration and high-glycemia diet, while protection from age-related macular degeneration features and low glycemia diet associated with increased *Bacteroidales*	(79)
*Faecalibacterium*, *Blautia*, *Roseburia*, *Lachnospira*, and *Ruminococcus*	Uveitis	Depletion of these butyrate producing bacteria are depleted in patients with uveitis compared to healthy controls	(73)
Streptococcaeae, Bacteroides, Bacteroidaceae, *Alistipes* and Erypsipelotrichaceae	Batten disease	*Cln1^R151X^ (infantile) mice had increased Streptococcaceae and decreased Bacteroides genus, while Cln2^R207X^ had increased Streptococcaeae, Bacteroides, Bacteroidaceae, Alistipes. Erypsipelotrichaceae* was increased for both compared to controls	(74)
*Bilophila*, *Alistipes*, and *Mucispirillum schaedleri*	Retinitis pigmentosa	*Bilophila*, *Alistipes*, and *Mucispirillum schaedleri* were most abundant in high-fat-diet in mice, which lead to greater retinal degeneration in *rd10* mice	(74)
*Firmicutes*, *Lactobacillus*, and Enterobacteriaceae	Retinopathy of prematurity	Preterm infants with retinopathy of prematurity associated with decreased Firmicutes and *Lactobacillus* with increased Enterobacteriaceae	(76)
*Blautia*, *Streptococcus*, *Faecalibacterium* and *Prevotella*	Dry eye syndrome	Increased abundance of *Blautia* and *Streptococcus* and decreased *Facecalibacterium* and *Prevotella* in patients with dry eye versus controls.	(78)
Gram-negative bacteria, butyrate-producing bacteria, lactate-producing bacteria, and methanogens	Vogt-Koyanagi-Harada disease	Patients were found to have gut microbiomes abundant with Gram-negative bacteria, while depleted of butyrate-producing bacteria, lactate-producing, and methanogen bacteria.	(80)

In conclusion, the intricate interplay between the GM and eye health unfolds through diverse communication channels, highlighting the potential of targeting the gut for novel therapeutic approaches in retinal diseases. From immune modulation and metabolite exchange to direct bacterial influences, the gut-eye axis presents a captivating avenue for future research and clinical applications. Understanding and harnessing this potent connection holds immense promise for revolutionizing the way we prevent and manage a spectrum of retinal ailments, offering hope for improved vision and well-being. Further investigation of the gut-eye axis will be critical in understanding how diet and metabolites play a role in disease. Metabolites of the greatest interest include SCFAs, butyrate, and BAs because of their implications in dysbiosis and inflammation.

## 4 Metabolites

**Figure 2:**
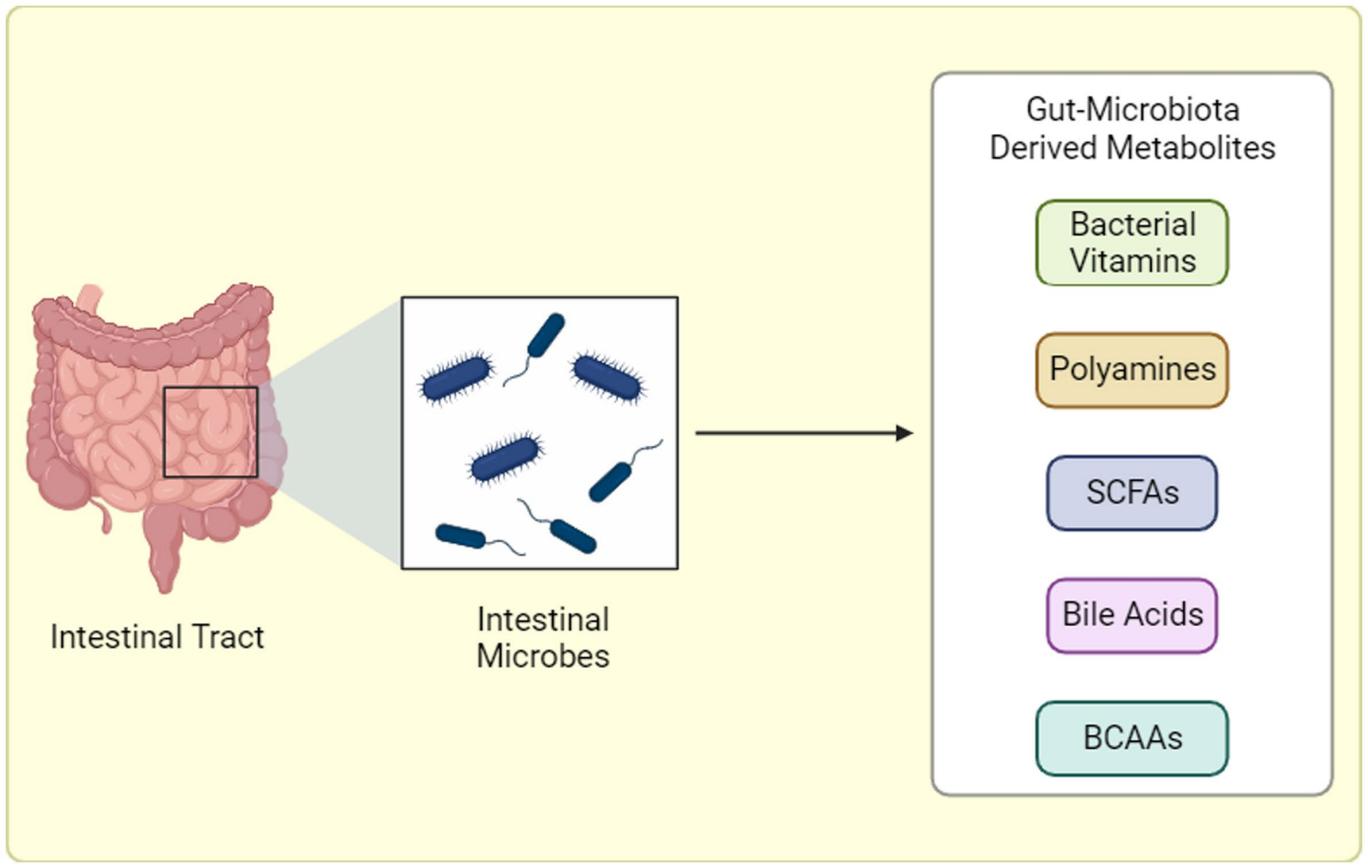
Gut Microbiota-Derived Metabolite Production. Gut microbiota generates a diverse array of metabolites through distinct pathways: (1) Dietary Fermentation: Here, bacteria “transform” ingested food components, like fibers, into bioactive molecules like short-chain fatty acids (SCFAs) or branched-chain amino acids (BCAAs). (2) Host-Microbial Collaboration: In this interplay, the host produces primary metabolites that gut bacteria refine into potent compounds. (3) Microbial Independence: Some bacteria possess the remarkable ability to “craft” specific metabolites, like trimethylamine-N-oxide (TMAO), entirely from scratch, independent of diet or host precursors. BCAAs: Branched-chain amino acids, SCFAs: Short-chain fatty acids, TMAO: Trimethylamine-N-oxide. “Created with BioRender.com”.

Metabolites are produced via various methods, including catabolism of food products in the gut (Figure 2). SCFAs are key metabolites produced by gut bacteria through the fermentation of dietary fiber. Specific bacteria, such as *Faecalibacterium prausnitzii* and *Roseburia* spp., are known to produce significant amounts of butyrate, a type of SCFA (81, 82). Butyrate has been extensively studied for its beneficial effects on gut health, including its anti-inflammatory properties and its role in maintaining the integrity of the intestinal barrier. Another important metabolite involved in GM communication are various BAs. Bile acids, produced by the liver and modified by the GM, not only aid in the digestion and absorption of dietary fats but also act as signaling molecules. They can influence various physiological functions, including lipid metabolism and glucose homeostasis (83–85). In states of dysbiosis, alterations in the production and metabolism of SCFAs and BAs can occur. This dysregulation may contribute to the development of certain pathological conditions, such as IBD and metabolic disorders.

### 4.1 Short-chain fatty acids

SCFAs are organic acids produced within the intestinal lumen. They are primarily produced by bacterial fermentation of undigested dietary carbohydrates (starches, celluloses, fibers, sugars) but also by dietary and endogenous proteins (86). There are three primary SCFAs: acetate, propionate, and butyrate. Butyrate is the primary source of energy for the epithelial cells within the colon (82). Acetate serves as a cofactor for enhancing the growth of other bacteria, and propionate can be converted to glucose in the intestine (87). SCFA absorption occurs through passive diffusion, as well as active transport by intestinal epithelial cells via sodium-coupled monocarboxylate transporter 1 (SMCT1) and proton-coupled monocarboxylate transporter 1 (MCT1) (88). SCFAs are downstream mediators of the GM anti-inflammatory activity and are fundamental to preserving immune homeostasis and functionality of the host immune system (89). SCFAs have anti-inflammatory properties through a variety of mechanisms. Microbial flora associated with the production of SCFA, such as *Bifidobacterium* spp., promote the production of anti-inflammatory cytokines, such as tumor necrosis factor (TNF) and IL-β. They also assist with the maturation of immune cells, promote IgA secretion, and possess antioxidant properties. SCFAs also reduce the production of cytokines by neutrophils and reduce macrophagic NF-κB signaling. The SCFA receptors within the colon are free fatty acid receptors and G protein-coupled receptors.

SCFAs have been shown to increase colonic Treg frequencies in the gut in mice, which are critical to regulating intestinal inflammation (90). In cases of intestinal breach, such as leaky gut or chronic inflammatory gut diseases, these SCFAs may travel in the bloodstream to other organs, including the eye. SCFAs can ameliorate immune-mediated ocular conditions partially by altering the migration of lymphocytes from the intestines, but once the SCFAs reach the eyes, they can also inhibit lipopolysaccharide (LPS)-induced intraocular inflammation (91, 92). Most often, metabolites and inflammation go hand in hand, as with DR, in which it is hypothesized that hyperglycemia and products of gut dysbiosis, such as uremic toxins, promote chronic low-grade inflammation, which impacts neovascularization (93).

Butyrate is a four-carbon SCFA that serves as the primary energy source for colonocytes and has many cellular functions impacting colonic health (82). Although, as discussed previously, the main three SCFAs are butyrate, propionate, and acetate, butyrate has the most anti-inflammatory properties and is the most studied within the literature as well (94). Butyrate has been implicated in a variety of health conditions, including ocular pathology. Once absorbed, 95% of butyrate is oxidized into ketone bodies for ATP synthesis (95). The 5% of butyrate not metabolized in the colon is transported to the liver and used as an energy substrate for hepatocytes, leaving little butyrate in systemic circulation (95). Butyrate specifically has been implicated in dysbiosis states of the GM and in several immune disorders, such as IBD, colorectal cancer, and type II diabetes (81). Recent studies, as detailed in Table 2. have also reported the efficacy of SCFAs in various retinal ailments. Similar decreases in butyrate due to the GM have been implicated as part of the possible pathogenesis of autoimmune Vogt-Koyanagi-Harada (VKH) disease (80). Transplantation of feces from patients with active VKH to mice undergoing EAU resulted in more severe manifestations of intraocular inflammation (80). Although both Behcet Syndrome and VKH disease cause uveitis involving GM dysbiosis, one study in China found specific changes in the GM for each etiology (80). Through metagenomic analysis, patients with VKH were found to be enriched in gram-negative bacteria in their gut; however, they were depleted with butyrate-producing bacteria, lactate-producing bacteria, and methanogens (80).

**Table 2:** Pharmacological effects of microbiome-related metabolites treatment on various ocular diseases.

Metabolite of Interest	Ocular Disease	Treatment and Dose	Findings	References
Butyrate	Retinopathy of prematurity	200 and 500 mg/kg/day (p.o)	Butyrate treatment reduced pathologic OIR changes compared to age-matched control.	(96)
Butyrate	Diabetic retinopathy	500 mg/kg b.w. (p.o.)	Sodium butyrate supplementation alleviates diabetic retinopathy through gut microbiota modulation and restoration of intestinal barrier function.	(97)
Butyrate	Age-related macular degeneration	Sodium Butyrate (NaB)-loaded nanoparticles/CS and pure NaBu at 34.4 μg /mLwere injected into the vitreous cavity	Chitosan-coated nanoparticles loaded with sodium butyrate offer a promising therapeutic strategy against choroidal neovascularization in wet AMD by demonstrating drug encapsulation, sustained release, ocular biocompatibility, and antiangiogenic activity.	(98)
Butyrate	Choroid neovascularization	1, 2.5, and 5 mM (i.v)	Sodium butyrate inhibits neovascularization by upregulating TXNIP, which then suppresses VEGFR2 expression and signaling pathways, leading to arrested cell cycle progression and ultimately hindering neovascularization.	(99)
Butyrate	Endotoxin-induced uveitis	500 mg/kg (i.p)	SCFAs cross the blood-eye barrier and modulate ocular inflammation and retinal astrocyte function, suggesting their potential as gut-derived immunomodulatory agents for eye diseases.	(91)
Butyrate	Ocular surface inflammation	0.5 mM of tributyrin (p.o.)	Butyrate, delivered through SCFA transporter SLC5A8, alleviates dry eye disease in mice by reducing inflammation and inhibiting Type I interferon signaling.	(100)
Butyrate	Intraocular bacterial Infection	10 μg/eye	Butyrate derivatives exhibit anti-bacterial and anti-inflammatory properties, promoting endophthalmitis resolution through autophagy-mediated bacterial killing and synergizing with antibiotics.	(101)
Butyrate	Uveitis	0.5 mM or 1 mM topically	Topical application of sodium butyrate at 0.5 mM moderately reduces the severity of endotoxin-induced uveitis in rats, suggesting its potential as a therapeutic agent for uveitis.	(102)
Butyrate and propionate	Autoimmune-mediated uveitis	150–300 mM propionate or 300 mM butyrate (p.o)	Butyrate treatment resulted in lower experimental autoimmune-Uveitis clinical scores compared to control.	(92)
TUDCA	Leber congenital amaurosis	500 mg/kg/b.w (Sc)	TUDCA helps to reduce endoplasmic reticulum stress, prevented apoptosis, and reduced cone degeneration.	(103)
TUDCA	Leber congenital amaurosis	500 mg/kg b.w. (Sc)	TUDCA is effective in reducing ER stress, preventing apoptosis, and preserving cones in Lrat−/− mice.	(104)
TUDCA	Retinal detachment	500 mg/kg b.w./day (Sc)	TUDCA treatment reduces cataract formation as a neuroprotective agent with decreased caspase activation.	(105)
TUDCA	Retinal degeneration	500 mg/kg b.w./day (Sc)	TUDCA preserved rod and cone structure and function by reducing oxidative stress and inhibiting caspase activity.	(106)
TUDCA	Retinitis pigmentosa	500 mg/kg b.w./day (Sc)	TUDCA was efficacious at on preserving rod and cone function and photoreceptor numbers.	(107)
TUDCA	Retinitis pigmentosa	500 mg/kg b.w./day (i.p)	TUDCA treatment resulted in decreased photoreceptor death and inhibited inflammation, resulting in a preserved retinal structure.	(108)
TUDCA	Retinal degeneration	500 mg/kg b.w./day (Sc)	Subcutaneous delivery of TUDCA inhibits the photoreceptor loss and visual impairments by modulating apoptosis and alleviating oxidative stress.	(109)
UDCA	Diabetic retinopathy	100 mg/kg b.w. (i.p)	ER stress and inflammation are suppressed by the protective effect of UDCA.	(110)
INT-777	Diabetic retinopathy	50 ng/μL, 5 μL/eye was injected into the vitreous cavity	INT-777 alleviated diabetes-induced retinal dysfunction such as vascular leakage and inflammation	(111)
TUDCA UDCA	Choroidal neovascularization	TUDCA 500 mg/kg/day. UDCA 100 mg/kg/day (i.p)	UDCA and TUDCA demonstrated anti-inflammatory action and suppressed laser induced CNV formation in rats.	(112)
TUDCA	Retinal degeneration	intravitreal injections of TUDCA-loaded PLGA MSs 5.05 ± 0.11 μg/mg	TUDCA slowed vision loss and retinal remodeling in an animal model of retinal degeneration, with neuroprotective effects in the posterior segment of the eye.	(113)
TUDCA	Retinal degeneration	500 mg/kg b.w. (i.p)	Neuroprotective effects of TUDCA help preserve rods and cones.	(114)
TUDCA	Retinal degeneration and retinal damage	500 mg/kg b.w. (Sc)	TUDCA profoundly suppressed apoptosis and preserved function and morphology of photoreceptor cells.	(115)
TUDCA	Optic nerve injury	100 mM TUDCA topically	TUDCA in combination with citicoline and neurotrophin-4 is the most effective way to protect retinal ganglion cells (RGC) after optic nerve crush injury.	(116)
TUDCA	Diabetic retinopathy	500 mg/kg bw–	TUDCA preserved visual and retinal function in a mouse model of diabetes, especially with early treatment.	(117)
UDCA	Diabetic retinopathy	15 or 30 mg/kg b.w. (i.p)	UDCA attenuates blood-retinal barrier (BRB) breakdown during DR development and reduces retinal inflammation.	(118)
UDCA	Diabetic retinopathy	100 mg/kg b.w. (Sc)	UDCA attenuates the retinal vascular abnormalities and retinal morphological changes in DR.	(119)
TUDCA	Retinitis pigmentosa	500 mg/kg b.w. (i.p)	TUDCA reduces anti-inflammatory action including the number and activation of microglial cells, and decreased microglial distribution in outer retinal layers.	(110)
UDCA	Wet age-related macular degeneration	125 mg/kg b.w. (p.o)	UDCA formulation was found to have inhibitory effects of choroidal neovascularization.	(120)
UDCA, GUDCA, or TUDCA	Retinopathy of prematurity	50 mg/kg/day(i.p)	UDCA can halt pathological neovascularization in the ischemic postnatal retina without affecting normal vascular growth or provoking systemic toxicity.	(121)
TUDCA	Cataract	500 mg/kg of b.w./day (i.p)	postpones sugar cataract formation by perhaps protecting the LECs from ER stress	(122)
TUDCA	Bardet-Biedl syndrome, retinal degeneration	500 mg/kg b.w. (Sc)	TUDCA ameliorated the obesity that accompanies retinal degeneration, and disrupt the pathway to apoptosis in the retina	(123)
TUDCA	Retinal ganglion degeneration	500 mg/kg/day (i.p)	TUDCA is neuroprotective in RGC by delayed cell loss and attenuating apoptosis	(124)
TUDCA	Diabetic retinopathy	250 or 500 mg/kg/d	TUDCA may be a potential drug for preventing and treating DR by protecting retinal vessels through decreased NOS, ICAM-1, NF-κB p65, and VEGF expression and reduced NO content.	(125)
TUDCA	Ocular Alkali Burn; choroidal neovascularization	400 mg/kg b.w. (i.p)	TUDCA treatment inhibits ER stress, retinal and neural inflammation, thus inhibiting CNV and inducing a protective effect in ocular alkali burn eyes.	(126)

TUDCA, tauroursodeoxycholic acid; UDCA, ursodeoxycholic acid; p.o., oral gavage; i.p, intraperitoneal; sc, subcutaneous; i.v., intravenous.

In a study by Chen et al., mice were injected with butyrate prior to intravitreal injection of LPS to study the effect of butyrate on intraocular inflammation (91). After the eyes were evaluated for 18 h, the authors noted that clinical signs of ocular inflammation in butyrate-treated mice were significantly milder than in control mice. The number of infiltrating cells (CD45+), a subset of monocytes/macrophages (CD45 + CD11b + Ly6c^hi^), and pro-inflammatory cytokines, such as IL-6 and CCL2, were also significantly reduced in the treated mice (91). Other SCFAs were studied, and they all inhibited IL-6 production in a dose-dependent manner, with butyrate being the most potent and acetate the least (91). The results of the study also suggest the existence of gut-eye cross talk and that SCFAs can cross the blood-eye barrier via systemic circulation. These findings also demonstrate the inhibitory roles of SCFAs on innate immunity, and their levels may inhibit or enhance intraocular inflammation (91). Oral propionate effectively reduced uveitis severity by boosting Treg and dampening effector T cells in the gut and lymph nodes. Notably, it hindered the migration of immune cells between the gut and the eye, suggesting a novel therapeutic approach. Further research on SCFA’s specific mechanisms and optimal doses could open the door to improved treatments for uveitis and potentially other inflammatory diseases (92). Oral supplementation with butyrate in diabetic mice also showed improved visual function compared to control diabetic mice (97). Oral butyrate also resulted in increased expression of Zonula occludens (ZO)-1and occludin proteins in the gut, representing increased gut barrier integrity. Furthermore, decreases in butyric acid, 4-methyl-valeric acid, and caproic acid associated with microbiota changes suggest a metabolic change exerted through the GM following oral butyrate supplementation. Another study sheds light on the potential of butyrate, in combatting intraocular bacterial infections like endophthalmitis. Butyrate derivatives injected into infected eyes effectively diminished bacterial growth and inflammatory responses, preserving retinal structure and function. The mechanism appears multi-pronged, involving both NLRP3-independent anti-inflammatory effects and enhanced bacterial killing through autophagy and antimicrobial peptides. Notably, butyrate synergized with antibiotics, suggesting its promise as an immunomodulatory and antibacterial co-therapy for improved visual outcomes in ocular bacterial infections (101). On a similar line, another study tested the potential of topical sodium butyrate as an alternative to corticosteroids in treating uveitis, an inflammatory eye disease. While not reaching the effectiveness of dexamethasone, butyrate at 0.5 mM dose showed a moderate but clinically relevant reduction in inflammation compared to untreated rats. Interestingly, its anti-inflammatory effect did not involve changes in measured cytokine levels, suggesting a distinct mechanism from corticosteroids (102).

The VEGF receptor 2 (VEGFR2) pathway plays a central role in orchestrating blood vessel growth. A study by Xiao et al. reveals a novel regulator of VEGFR2: TXNIP, whose expression is significantly enhanced by sodium butyrate (NaBu). Interestingly, NaBu potently inhibited neovascularization in various models, suggesting its anti-angiogenic properties may work through the TXNIP-VEGFR2 axis. This opens exciting avenues for developing new therapies targeting this critical pathway in diseases characterized by excessive blood vessel growth (99).

It is known that a function of intestinal epithelium derived butyrate is to help maintain the intestinal barrier by modulating goblet cell expression of mucins and goblet cell differentiation (127). One study hypothesized butyrate produced in the gut also has a direct effect on the differentiation or maintenance of conjunctival goblet cells, lending to its role in dry eye disease (128). In a pharmacologic study, butyrate was shown to modulate the inflammatory responses at the ocular surface and, when delivered intragastrically, attenuated ocular surface disease in a dry eye disease mouse model through SCFA transporter SLC5A8 (100). *In-vitro* experiments showed the reduction of Tumor Necrosis Factor alpha (TNF alpha) expression in corneal epithelial cultures when pre-treated with phenylbutyrate. Cultures from Slc5a8 knockout mice showed no response to pre-treatment with phenylbutyrate. Furthermore, the anti-inflammatory effect of butyrate *in vivo* was shown to partially require SLC5A8 (100).

The role of the GM in the gut and retinal brain barrier has been shown through experiments elucidating the improved integrity in both organs and after probiotic use in mice, potentially playing a role in the severity of DR (129). Alongside butyrate, other compounds and medications have been shown to impact the microbiome via modulation of SCFAs. Fenofibrate is an FDA-approved lipid-lowering medication used to treat hypertriglyceridemia, primary hypercholesterolemia, and mixed dyslipidemia (130). High-fat diets (HFD) significantly decrease levels of SCFA in the serum and retina, which fenofibrate supplementation mitigates. Fenofibrate can also decrease inflammation within the retina by reducing activation of microglia and Muller cells, inhibiting toll-like receptor 4 expression (TLR-4), and decreasing the expression of inflammatory cytokines in the eye, such as TNF-alpha, IL1beta (Interleukin-1 beta), and IL-6 (Interleukin 6) (131). One study speculated the potential for fenofibrate to attenuate HFD-induced inflammation in the retina through suppressing lipopolysaccharide (LPS)-TLR4/inflammatory cells-activation of the NF-kB and JNK signaling pathways-inflammatory cytokines (131). Thus, fenofibrate’s role in modulating levels of SCFAs can be a candidate for future pharmacologic investigation, as well as an avenue to better understand SCFAs’ inflammatory effects. Increases in butyrate-producing bacteria, such as *Lachnospiraceae*, after oral omega-3 fatty acid supplementation support potential gut-microbiome-focused approaches to disease. Coupled with epidemiological studies that show potential protective effects of consumption of omega-3 fatty-acid-rich fish against AMD, the potential for butyrate as a pharmacologic target in AMD is tantalizing (94, 132). Of interest, Reis et al. designed sodium butyrate-loaded nanoparticles coated with chitosan for enhanced ocular biocompatibility. In AMD models, these nanoparticles effectively inhibited neovascularization, highlighting their potential as a targeted therapeutic strategy against abnormal blood vessel growth (98). Our research group recently reported on the pharmacologic application with butyrate in the oxygen-induced retinopathy (OIR) model, which replicates pathologic neovascularization in mice similar to ROP (96). Mice pups subjected to OIR had significantly decreased butyrate-producing bacteria in cecal samples compared to mice pups on room air (RA). Furthermore, OIR pups treated with butyrate via oral gavage had significantly decreased neovascular tufts and areas of avascular retina, compared to age-matched control mice. Butyrate demonstrated significantly decreased extravasation on leakage assays and restoration of ZO-1 levels, supporting the role of butyrate in preserving the blood-retina barrier.

**Figure 3:**
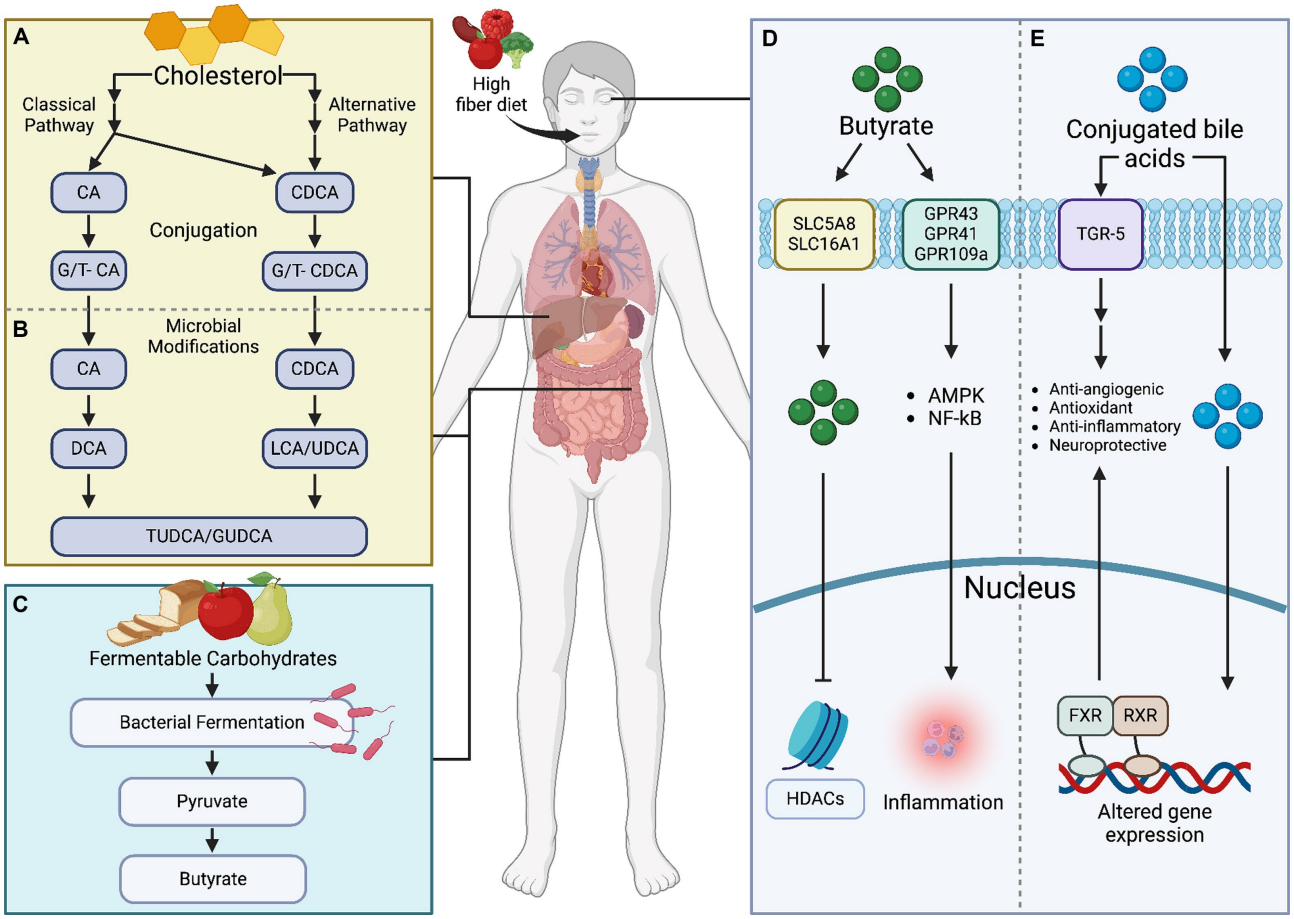
Microbial metabolites, specifically bile acids and butyrate, exert significant influence on retinal health. **(A)** Bile acids, produced through cholesterol metabolism, impact retinal inflammation, vascular function, and oxidative stress. Meanwhile, **(B)** butyrate, a short-chain fatty acid, modulates **(C–E)** immune responses and neuronal survival within the retina. Understanding these metabolites’ roles is crucial for developing targeted therapies to mitigate retinal pathologies. Created with BioRender.com.

In conclusion, SCFAs, particularly butyrate, emerge as potent regulators of ocular health through diverse mechanisms (Figure 3). From dampening inflammation and modulating barrier integrity to influencing angiogenesis and bacterial killing, SCFAs hold immense promise for novel therapeutic approaches in various retinal diseases. Research exploring their gut-eye communication pathways, optimal delivery methods, and synergistic potential with other interventions paves the way for revolutionizing our understanding and treatment of these debilitating conditions. As we delve deeper into the intricate world of the GM and its influence on the eye, the therapeutic horizon continues to expand, offering hope for improved vision and well-being for individuals suffering from retinal diseases.

### 4.2 Bile acids

BAs are produced in hepatocytes and transported to the gallbladder through bile canaliculi. They are further modified in the intestinal lumen by the GM through hydroxylation, deconjugation, oxidation, and epimerization (133, 134). 95% of the total BAs (unconjugated and conjugated) are reabsorbed and returned to the liver via enterohepatic circulation (135, 136). Of the 95% reabsorbed, only 10% of BAs reach systemic circulation, therefore, BA synthesis in the liver is crucial (135). The 5% that is not reabsorbed is excreted in the feces (135, 136). BAs are hormones with unique chemical compositions that contribute to their anti-inflammatory and antioxidant effects. They serve many different roles in the body’s digestive and metabolic systems, which has long been appreciated.

BAs maintain liver cell viability, assist with cholesterol catabolism, lipid digestion, and absorption, and stimulate bile flow and biliary phospholipid secretion (83–85). They are produced in hepatocytes through two major pathways: the classical and alternative pathways (83). In the classic pathway, cholesterol is converted to 7α-hydroxycholesterol by the rate-limiting enzyme CYP7A1 (cholesterol 7 alpha-hydroxylase A1) (135, 137). 7α-hydroxycholesterol is ultimately converted to cholic acid (CA) by a sterol 12α-hydroxylase (CYP8B1) or converted to chenodeoxycholic acid (CDCA) spontaneously (135, 137). In the alternate pathway, cholesterol is first converted to 27-hydroxycholesterol by CYP27A1 (cytochrome P450 Family 27 Subfamily A Member 1) and then converted to CDCA (135, 138, 139).

BAs are utilized in traditional Chinese medicine (TCM) (140–142), as bear bile is used to remove toxins from the body, stop convulsions, and improve vision (140–142). BAs have also been used therapeutically for liver disease and biliary cirrhosis in both TCM and modern Western medicine (141–143). The discovery of their signaling properties and ability to regulate the activity of genes through the orphan nuclear receptor, farnesoid X receptor (FXR), has generated more interest in their function in homeostasis and metabolism (144). Most of the earlier studies on the pharmacologic use of BAs have focused on the liver because it is the primary site of BA production, but recent studies have also identified synthesis within extrahepatic sites, such as the brain and retina (135, 145). Production in these sites relies on the alternate synthesis pathway, which plays an important role in retinal cholesterol and BA metabolism (135, 146).

In the retina, BAs act as signaling molecules and can activate the FXR and TGR5 receptors; however, the literature is limited, and TGR5 expression has only been reported in the adult retina (147). Interestingly, studies with mice have shown the protective effects against DR through intermittent fasting, which exerts its effects through microbiome restructuring and TGR5 activation (148). Through activation of the TGR5 receptor, BAs like ursodeoxycholic acid (UDCA) and tauroursodeoxycholic acid (TUDCA) can also reduce acellular capillaries, inflammation, and the number of macrophages, leukocytes, and activated microglia (148). In the last decade, studies have proposed to prove the usefulness of BAs to treat ocular diseases (146, 149). Recent studies have shown BAs are protective in neurodegenerative diseases, including several ocular afflictions, including Leber’s congenital amaurosis (103, 104), retinal detachment, cataracts, retinitis pigmentosa, diabetic retinopathy, choroidal neovascularization, retinopathy of prematurity (ROP) (Table 1). The mechanism of action of UDCA and TUDCA is unclear; however, it is hypothesized to involve the suppression of caspase-dependent and caspase-independent apoptosis by inhibiting the release of apoptosis-inducing factor (AIF) from the retinal pigment epithelium (RPE), photoreceptors, and RGC (145, 150). Paradoxical effects and an incomplete understanding on cancer cell proliferation from BAs have added notable caution to approaching these metabolites as therapeutic targets (151). Nevertheless, the potential for ocular applications remains promising.

Several preclinical studies suggest promising roles for TUDCA in protecting and preserving vision in retinal degeneration models. Boatright et al. observed significant preservation of photoreceptor cells, function, and morphology in mice with light-induced retinal degeneration after systemic injections of TUDCA (500 mg/kg) (115). Similarly, another study showed that both bilirubin and TUDCA reduced oxidative stress and protected photoreceptors in mice exposed to bright light or carrying a retinal degeneration mutation (106). Focusing on specific retinal cell types, Tao et al. found that subcutaneous TUDCA delivery effectively preserved cone photoreceptors and visual function in a chemically induced rodent model of retinopathy (109). Fernández-Sánchez et al. demonstrated that weekly TUDCA injections preserved photoreceptor structure and function in another RP model, highlighting its potential as a long-term neuroprotective therapy (114). Additionally, in a model of retinitis pigmentosa, Fernández-Sánchez et al. reported that slow-release TUDCA microspheres injected into the eye provided sustained protection for photoreceptors, synaptic connections, and retinal function (113). However, some studies indicate strain-specific effects and varying degrees of efficacy depending on the delivery method and treatment duration. Another study observed partial preservation of function and structure in one strain of rd1 mice with rapid retinal degeneration, but only maintained structure in the other (124). This suggests further research is needed to optimize TUDCA treatment for different forms of retinal degeneration. Overall, these preclinical studies paint a promising picture for TUDCA’s potential in protecting vision against retinal degeneration.

Several studies highlight the promising potential of TUDCA in protecting vision and delaying retinal degeneration in animal models of RP. Phillips et al. observed significant preservation of photoreceptor structure and function in rd10 mice treated with TUDCA (500 mg/kg every 3 days) from postnatal day 6 to 30, demonstrating its efficacy in early stages of the disease (107). Similar effects were seen in Rpgr knockout mice, where TUDCA treatment prevented photoreceptor degeneration and suppressed microglial activation, suggesting its potential against specific RP mutations (108). Noailles et al. further explored the neuroprotective mechanisms, finding that TUDCA reduced microglial activation in a P23H rat model of RP (500 mg/kg weekly injections), offering an additional therapeutic avenue (152). These studies, with their different treatment durations and RP models, converge in suggesting TUDCA as a promising candidate for future RP therapies, warranting further research to optimize its application and efficacy in humans.

Growing evidence suggests potential for UDCA and its derivatives in mitigating DR progression. Studies exploring diverse mechanisms highlight UDCA’s ability to protect retinal microvasculature and preserve visual function. Chung et al. demonstrated UDCA’s effectiveness in reducing pericyte loss in diabetic mice (500 mg/kg), potentially delaying BRB breakdown (110). Zhu et al. identified TGR5 receptor activation as a promising therapeutic target, with its agonist attenuating DR via RhoA/ROCK signaling suppression (111). Another study observed early TUDCA treatment (50 mg/kg) preserving visual function and retinal structure in a mouse model, emphasizing the importance of timely intervention (117). Ouyang et al. revealed UDCA’s anti-inflammatory properties in DR, reducing retinal inflammation and reversing BRB breakdown (15 mg/kg and 30 mg/kg) (118). Additionally, Shiraya et al. demonstrated UDCA’s ability to rescue retinal vasculature and reduce edema in an antibody-induced pericyte depletion model (25 mg/kg) (119). Finally, Wang et al. explored TUDCA’s protective effects on human retinal microvascular endothelial cells and DR rats, suggesting its potential in preventing and treating DR by downregulating inflammatory mediators (5.0 μM to 125.0 μM *in vitro* and 250 mg/kg and 500 mg/kg *in vivo*) (153). These preclinical findings paint a promising picture for UDCA and its derivatives as potential therapeutic options for DR, warranting further clinical investigation to translate these benefits into effective patient care. Specific BAs, Taurolithocholic acid (TLCA) and TUDCA, have also been shown as independent predictors for DR in metabolic profiles of patients with Type 2 diabetes mellitus (T2DM) (154).

Oral delivery of UDCA has shown promise in treating choroidal neovascularization (CNV) associated with wet AMD. Maharjan et al. developed an aqueous oral UDCA formulation with improved bioavailability and demonstrated its efficacy in a mouse model. This formulation effectively suppressed CNV growth and improved retinal function after oral administration (120). Another study provided further evidence for UDCA’s potential, showing that systemic administration of UDCA (500 mg/kg) and its taurine conjugate TUDCA (100 mg/kg) significantly reduced CNV size and leakage in a laser-induced rat model (112). These studies suggest that oral UDCA formulations warrant further investigation as a non-invasive and potentially cost-effective alternative to current intravitreal injections for managing CNV and WAMD.

Photoreceptor cell death and retinal detachment are associated with numerous ocular diseases (155), and a study by Mantopoulos et al. demonstrated that TUDCA preserved photoreceptor survival by reducing ER and oxidative stress (105). TUDCA has also been shown to prevent cataract formation in galactosemic rats and slow the progression of retinal degeneration by inhibiting the unfolded protein response (UPR)-dependent pathway and reducing superoxide radicals, respectively (122). TUDCA has emerged as a promising neuroprotective agent against retinal ganglion cell (RGC) loss. Gómez-Vicente et al. demonstrated that systemic TUDCA administration (30 mg/kg) in NMDA-induced retinal injury in rats preserved RGC density and improved electroretinogram amplitudes, suggesting its potential for treating diseases involving RGC degeneration (125). Furthermore, TUDCA’s protective effects extend to cataract formation. Mulhern et al. reported that oral TUDCA treatment (300 mg/kg) in galactosemic rats effectively reduced lens epithelial cell death and delayed cataract development, possibly by mitigating endoplasmic reticulum stress and oxidative stress (122). These findings highlight the multifaceted neuroprotective and anti-cataract properties of TUDCA, warranting further investigation of its therapeutic potential in various ocular diseases.

Pharmacologic application has involved UDCA, that reduces retinal inflammation by reducing pericyte depletion, expression of inflammatory cytokines, such as IL-1β and IL-6, and expression of angiogenic factors and inflammatory mediators, such as ICAM-1 (118). In our previous study, OIR mouse pups showed attenuated pathologic neovascularization, decreased OIR-induced BRB dysfunction, and decreased levels of oxidative stress when treated with UDCA (121). In the OIR model, our research group demonstrated the protective role of BAs, such as UDCA, within the GM for the pathogenesis of ROP. Cecal samples from OIR mice compared to RA mice showed increased levels of unconjugated BAs. Furthermore, the microbiota in OIR mice consisted of lower levels of bile-salt hydrolase (BSH)-producing phyla in compared to control mice (156). Further studies in retinal BA signaling showed compromised FXR signaling in OIR mice, exacerbated OIR pathology in FXR knockout mice, and amelioration of OIR with OCA, an FXR-specific agonist (157).

Additionally, TUDCA has emerged as a promising therapeutic agent for various retinal diseases treating a wide range of retinal diseases, including ocular alkali burns, and glaucoma due to its anti-inflammatory, neuroprotective, and regenerative properties. In a mouse model of ocular alkali burn, Huang et al. demonstrated that systemic TUDCA administration (400 mg/kg) effectively suppressed corneal and retinal inflammation, protected RGC from apoptosis, and promoted corneal re-epithelization (126). Another study reported that TUDCA treatment (subcutaneous injections, twice a week) preserved photoreceptor function and structure in two different mouse models of retinitis pigmentosa, Bardet-Biedl syndrome mice, and rd10 mice (123). Kitamura et al. investigated the neuroprotective effects of TUDCA in a rat optic nerve crush model. They found that topical application of TUDCA, alone or in combination with other neurotrophic factors, significantly protected RGC from degeneration (116).

BAs have emerged as promising therapeutic agents for various ocular diseases due to their multifaceted roles in digestion, metabolism, and signaling. BAs exert neuroprotective and anti-inflammatory effects, making them suitable candidates for treating photoreceptor degeneration, diabetic retinopathy, and ROP (Figure 3). Despite the encouraging preclinical findings, further research is necessary to elucidate the precise mechanisms of BA action in the eye, optimize the route of administration and dosage, and ensure long-term safety and efficacy for clinical translation. Understanding the crosstalk between BAs, the GM, and retinal BA signaling pathways will be crucial for developing novel therapeutic strategies targeting ocular diseases.

### 4.3 Other metabolites

While we summarize the crucial roles of SCFAs and BAs in the body and how they relate to ocular health, it should also be noted that the gut microbiota itself affects the metabolism of amino acids that in turn impact ocular health. Of note, tryptophan has also been linked to the gut-brain axis (79). In this study, mice fed a high-glycemic diet had low levels of the metabolite serotonin compared to mice fed a lower-glycemic diet. The high glycemic diet resulted in many AMD features, such as photoreceptor degeneration, hypopigmentation, and atrophy linked to the accumulation of advanced glycosylation end products. It was found that in mice with AMD-like changes, there was an inverse relationship between serotonin and frequency of AMD features. Since serotonin derives from tryptophan, an increased number of spores forming bacteria in the gut to create more serotonin may be protective in cases of AMD (79). The study also found that microbiota in the *Clostridiales* order were associated with AMD features, while the *Bacteroidales* order was associated with protection (79). Other metabolites investigated include branched-chain amino acids (BCAAs) and glutamate. BCAAs are key components in glucose and protein metabolism, and their decomposition can be altered through dysbiosis. This ultimately promotes oxidative stress responses, and raised serum levels of BCAAs may predict the development of T2DM. Specifically, levels of leucine, isoleucine, and valine in BCAA metabolism were found in studies to be increased in the serum of DR patients and in the diabetic rat retina (158). In one study, mice were fed a short-term diet with a reduction of BCAAs, and they had improved metabolism of white adipose tissue and intestinal microbiota composition with a lowered postprandial insulin secretion (159). Catabolism of BCAAs provides nitrogen for the synthesis of glutamate, and glutamate plays an important role in DR neurodegeneration as it is the major excitatory neurotransmitter in the CNS and retina. Branched chain amino acids (BCAAs) are associated with glutamate toxicity contributing to retinopathy (160), especially in DR and AMD. It has been found that BCAA supplements play a neuroprotective role by enhancing retinal ganglion cell survival, in addition to inflammatory and oxidative stress protective effects (160). BCAAs from protein diets are absorbed by the intestine, thus any dysregulation in absorption through altered gut microbiomes or intestinal malabsorption syndromes could be implicated in lower BCAA levels and susceptibility to retinopathy.

Within the retina, glutamatergic synapses connect its fundamental functional cells, such as photoreceptors, bipolar cells, and retinal ganglion cells. Studies found that increased glutamate in the retina activates ionotropic glutamate receptors in excess, mainly the NMDAR, resulting in uncontrolled intracellular calcium responses and cell death (161). Other studies have tried to confirm the correlation between intake of glutamate with the incidence of DR and have not found significant results, so more studies need to be done before glutamate can be used for screening of DR or used as a measure of treatment response. High levels of the glutamate, a neurotransmitter derived from the amino acid glutamic acid, triggers a neurotoxic cascade in the retina (162, 163). This can be explained by glutamate’s activation of NDMA (N-nitrosodimethylamine) receptors causing retinal ganglion cell death in DR, glaucoma, and retinal ischemia (162, 163). Other amino acids, such as arginine and lysine, have also been previously summarized for their impacts in DR, AMD, and ROP (164), though most notably it is glutamine and arginine that appear to be biomarkers of early retinopathy diagnosis (164). Glutamate enters the TCA cycle after being absorbed by the intestines and is converted to other amino acids such as arginine. Dietary glutamate is also used an energy source by the intestines (165). By decreasing glutamate levels through receptor inhibition, including NMDA receptor modulation, diseases like DR can improve (162, 164). Metabolism of amino acids due to gut microbiota, in addition to SCFAs and BAs, should be considered for their important effects on ocular health.

It has been long established that the microbiota has the ability to produce vitamins, notably vitamins B and K. Many species harbored in the gut microbiome have been shown to have potential in synthesizing such vitamins (166). However, more recently, there has come to light a role for the microbiota in regulating host metabolism of Vitamin A, and further, they have been able to directly metabolize vitamin A (167). Also described is a relationship between the gut microbiota and vitamin D, with the gut microbiota playing a role in vitamin D metabolism (168). Outside of gut microbe effect on metabolism of vitamins, there seems to be an emerging role for vitamins as modulators of the gut microbiota, which could help to play a role in the clinical applications of gut microbiota (169). For example, altering one’s intake of different vitamins could work to shape their gut microbial community.

Free fatty acids (FFA’s) represent another group of molecules that can be affected by GM in various manners. Some GM are capable of metabolizing dietary fats directly, ultimately converting them into FFAs (170). For this reason, GM dysregulation and its subsequent effect on FFA concentration is likely to play a role in various ocular pathologies. Although, the specific role of FFA’s in multiple ocular pathologies has recently become the subject of much speculation. Altered FFA levels are often seen in chronic diseases and ocular pathologies that present as comorbidities (171). T2DM, for example, is often associated with increased FFA levels, and it has been hypothesized that FFA’s may facilitate a hyperperfusion response that results in deteriorated microvascular function (172). Furthermore, low grade chronic inflammation (LGCI) is a pro-inflammatory state associated with many chronic conditions and ocular diseases that present as comorbidities, including diabetes mellitus linked retinopathy. LGCI, when left unchecked, can eventually lead to a depressed anti-inflammatory response. Long-term diabetes mellitus can also reduce blood-retinal barrier integrity, which leads to free radical and pro-inflammatory molecule release, polyunsaturated fatty acid pathway dysregulation, and increased VEGF levels (171).

With the shift toward an inflammatory and oxidative state, FFA’s have been investigated as a potential therapeutic agent in modulating inflammation within the context of ocular disease. Long term exposure to a hyperglycemic environment culminates in marked oxidative stress levels that can lead to free radical formation and apoptosis, deteriorating retinal microvasculature (173). Omega-3 polyunsaturated fatty acids (ω-3 PUFA’s) represent a FFA that has been found to elicit an anti-inflammatory and antioxidant response in multiple ocular pathologies, including diabetic retinopathy. Due to high photoreceptor demand for PUFA’s, it is recommended that fish oil supplements be taken daily as a valuable source of ω-3 PUFA’s (173).

Ultimately, the notion that the modulation of free fatty acids by the GM plays an important role in ocular health appears to be well substantiated based on currently available data. FFAs have been clearly linked to multiple forms of ocular pathogenesis, including but not limited to deteriorated microvascular function, a pro-inflammatory state, and oxidative stress (171–173). FFA’s, such as ω-3 PUFA, also represent a potential therapeutic agent in the treatment of oxidative stress in ocular disease (173). As both a direct and indirect modulator of FFA levels, the GM should be considered an important factor in FFA dysregulation. Although, more research is required to elucidate the complex nature of the relationship between the pair in the context of eye disease.

### 4.4 Detrimental potential of metabolites

Bacteroidetes, a prominent phylum within the gut microbiota, harbors Gram-negative bacterial species that elicit immune-mediated responses via components of their cell wall, particularly lipopolysaccharide (LPS) and flagellin. These responses are initiated when pattern recognition receptors (PPRs) of the innate immune system detect these bacterial products. For instance, the sensing of flagellin by dendritic cells triggers the production of IL-22 by innate lymphoid cells (ILCs), as demonstrated in studies like (174). Additionally, variations in toll-like receptor (TLR) 4, responsible for recognizing LPS and potentially leading to changes in the trabecular meshwork, have been linked to primary open-angle glaucoma (POAG), as discussed in the review article by (175). Moreover, experimental studies utilizing animal models of glaucoma have revealed the detrimental impact of exogenously administered LPS on axons and neurons, as seen in investigations such as those by Napolitano et al. (18). Notably, these experiments also showed that exogenous LPS exacerbated photoreceptor loss and impaired retinal function in dystrophic P23H rats, as reported in studies like (176). While previous sections delve into metabolites that can positively modulate ocular health, the role of LPS underscores the potential for microbiota-derived substances to have adverse effects on ocular tissues.

## 5 Clinical trials

There are countless animal studies looking at the role of the GM and its relation to certain diseases, and over the past decade, the number of human clinical trials has quickly grown as well. UDCA was one of the first BA’s to be widely utilized to treat primary biliary cirrhosis, and the research on the compound has continued. In the PEGASUS-D study in Korea, adults with a diagnosis of gastric cancer and who underwent gastrectomies were enrolled and randomly assigned to receive different dosages of UDCA or placebo. Patients were followed over a 12-month period, and it was found that administration of UDCA significantly reduced the incidence of gallstones after gastrectomy in patients with gastric cancer (177). Recently, studies have looked at SCFA’s role in neurological disorders as well. In one study on ALS, treatment with TUDCA was associated with a slower deterioration of function; TUDCA has no known symptomatic modulation on muscle strength or motor function, so this observation may potentially reflect a disease-modifying effect. Similarly, high-dose UDCA was given to patients with early Parkinson’s Disease (PD), and midbrain P-MRS demonstrated improved ATP hydrolysis with a possible improvement in cadence and other gait parameters as well (178). Incredible advancements have been made with GM research and its clinical applications, but there is still much to be studied. Despite the absence of clinical trials, substantial preclinical data support the exploration of microbiome-related metabolites as promising candidates for managing retinal diseases.

Bile acid derivatives, while therapeutically promising as our lab’s and previous work has shown, are not as well-researched as other gut-health enhancing domains like prebiotics or probiotics. This could be due to multiple reasons. One reason is that the function of bile acid derivatives in disease relevance is novel, with the gut microbiome and metabolomics only recently coming to interest in contributing to overall health. Another is that there must be substantial preclinical evidence before advancement to clinical trials. While SCFAs have been implicated in diseases like IBD, there has been much more volume of research in other domains not related to the specific organ system of the eye. Finally, clinical need is another factor contributing to lack of bile acid derivative clinical trials. We anticipate an increase in SCFA and BA clinical trials in the future as more research and animal trials support the need for clinical trials.

## 6 Conclusion

The GM plays a crucial role in human health and disease, and the gut-eye axis is a relatively novel avenue needing further research to understand functions and potential clinical applications, even in Ophthalmology, where the paradigm of organ sterility is constantly being challenged. The GM and its metabolites have emerged as a major player for many pathologies of the eye. This review highlights the importance of understanding the function of the GM and its metabolite production, particularly butyrate and BAs. The bidirectional relationship between the GM and its metabolites is useful in these therapeutic opportunities; however, it should be explored further in hopes to better understand the relationships between GM, metabolites, and the eye. Our ongoing studies are focused on exploring microbiome related metabolites as a potential therapeutic strategy in retinal disease. Hence, focusing on microbiome-related metabolites over the broader approach of fixing the entire microbiome offers a more targeted and precise intervention. This targeted strategy is expected to provide a more nuanced understanding of the microbiome ecosystem. We are hopeful that this focused strategy, will not only enhance precision but also offer a more efficient pathway toward achieving desired outcomes in optimizing gut health and its implications in retinal diseases.

## Author contributions

YN: Writing – original draft, Writing – review & editing. JRZM: Writing – original draft, Writing – review & editing. NMR: Writing – original draft, Writing – review & editing. TB: Writing – original draft, Writing – review & editing. AO: Writing – review & editing. RNJ: Conceptualization, Supervision, Writing – review & editing. MCT: Conceptualization, Supervision, Writing – original draft, Writing – review & editing.
